# Treatment of hepatic carcinoma by low-frequency ultrasound and microbubbles: A case report

**DOI:** 10.3892/ol.2014.2812

**Published:** 2014-12-18

**Authors:** ZHI-YONG SHEN, MING-FENG WU, YI-XIN ZHANG, KANG SHEN, GAN-LIN XIA

**Affiliations:** Department of Radiology, Nantong University Affiliated Nantong Tumor Hospital, Nantong, Jiangsu 226361, P.R. China

**Keywords:** hepatic tumor, low-frequency ultrasound, therapy, microbubbles, cavitation

## Abstract

*In vitro* and *in vivo* studies have identified that low-frequency ultrasound (US) and microbubbles (MBs) mediate tumor inhibitory effects. However, the application of US in the clinical setting remains unclear. The aim of the present study was to investigate the clinically therapeutic effect of 20 kHz US in combination with MBs for the treatment of hepatic carcinoma. A 71-year-old male with a hepatic malignant tumor was admitted to Nantong University Affiliated Nantong Tumor Hospital (Nantong, China). The patient was subsequently sonicated with 20 kHz US and MBs over a period of five days. The low-frequency US parameters were set at 20 kHz, 2 W/cm^2^, duty cycle 40% (on 2 sec, off 3 sec) for a duration of 5 min each day for a total of five days. Computed tomography (CT), contrast-enhanced US (CEUS) and carbohydrate antigen 19-9 (CA19-9) tests were performed to evaluate the therapeutic effects. Although the tumor size increased marginally on CT from 5.4 to 5.6 cm after US treatment, the intensity and enhanced-areas on the CT scans and CEUS decreased. The abdominal lymph node decreased in size, from 2.2 to 1.9 cm, and CA19-9 levels decreased from the pretreatment value of 2,007 to 734 U/ml. Therapy with low-frequency US combined with MBs may exhibit an antivasculature effect and may be used as a palliative treatment for patients with unresectable hepatic malignant tumors.

## Introduction

Ultrasound (US) is a diagnostic tool used clinically, however, this technique is also used therapeutically. US therapy has been clinically investigated as a tumor treatment with a particular focus on the thermal effects generated by high-intensity focused US (1). However, few clinical studies have investigated the non-thermal mechanisms induced by low-frequency US, such as acoustic cavitation (2–4). Ultrasonic transducers with a frequency within the kHz range produce the most evident cavitation effects (5). *In vitro* cavitation studies have demonstrated the ability of US to induce morphological changes, plasma membrane wounding (6) and cell lysis (7). A previous study showed that cavitation induced by 24 kHz US resulted in a loss of viability of cellular membranes, apoptotic death and immediate necrosis (8). Furthermore, *in vivo* study on organs that contain air, such as the lungs, has demonstrated that US may cause tissue hemorrhage (9). *In vivo* cavitation may alter the permeability of individual cells, leading to improved delivery of genes and drugs (10). Inertial cavitation has also been found to lead to marked destructive effects *in vivo*, as shown by reduced rates of tumor growth (11,12) and increased survival time of mice (13). Our previous findings indicated that low-intensity US with microbubbles (MBs) may exhibit anti-angiogenic effects on subcutaneous tumors in nude mice (14,15). *In vitro* and *in vivo* bioeffects of kHz-range US have been reported previously. However, the application of US in combination with microbubbles in a clinical setting remains unclear. The aim of the present study was to investigate the clinically therapeutic effect of 20 kHz US combined with MBs for the treatment of hepatic carcinoma.

## Case report

### Clinical data

**Figure 1:**
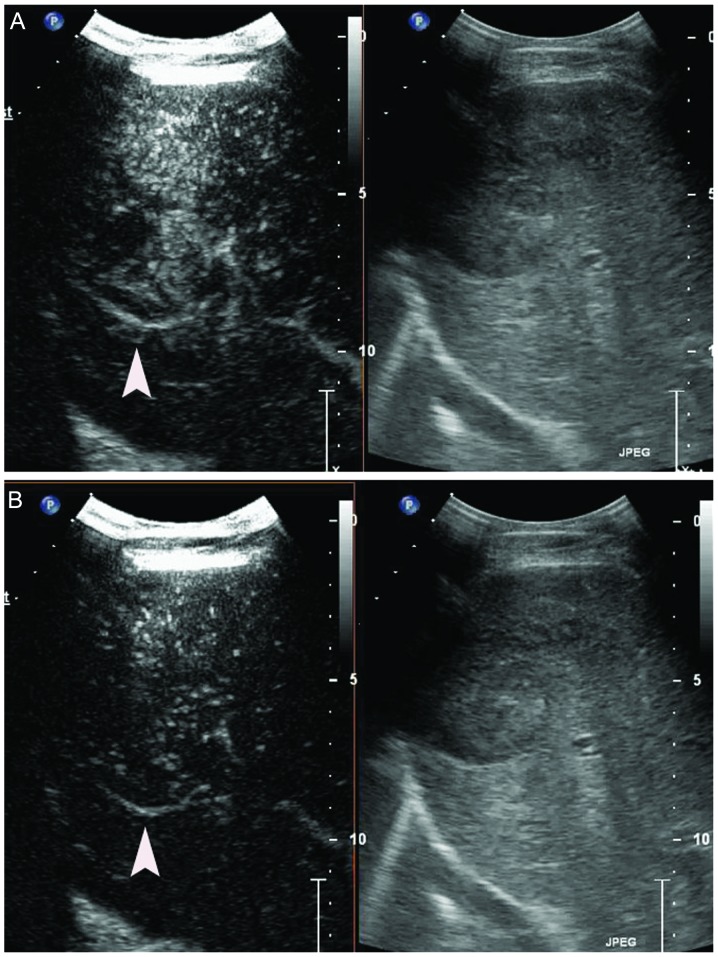
CEUS showing that the intensity of the tumor and enhancement area decreased after five days of treatment with 20 kHz US combined with MBs. (A) CEUS of the liver obtained during the hepatic arterial phase showing the tumor (arrow) as a heterogeneously enhancing mass in the hepatic dome 24 sec after SonoVue was injected. (B) After five days of treatment, a CEUS scan of the liver obtained during the hepatic venous phase at the same level as (A) showing that the tumor (arrow) enhancement intensity decreased when compared with the enhancement areas in (A). US, ultrasound; MBs, microbubbles; CEUS, contrast-enhanced ultrasound.

**Figure 2:**
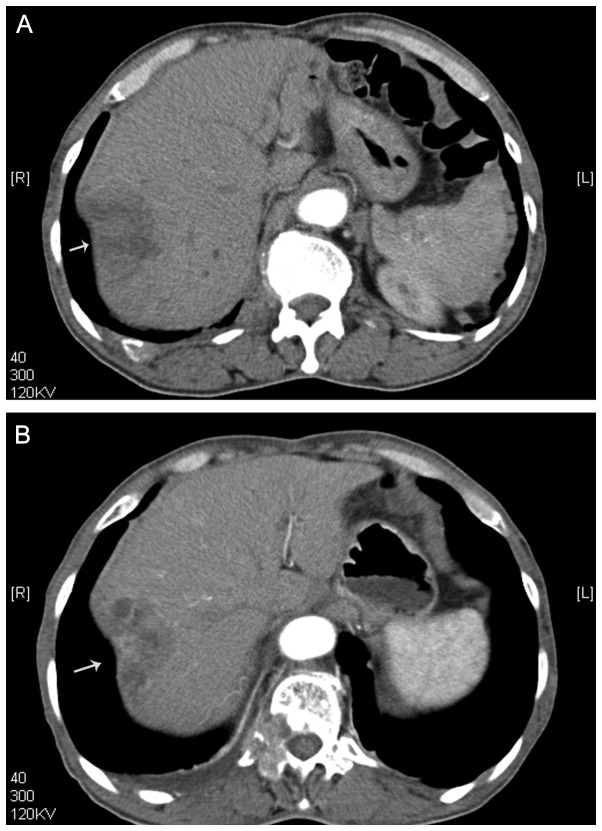
CT scan showing that the intensity of the tumor and enhancement area decreased after five days of treatment with 20 kHz ultrasound combined with microbubbles. (A) Axial contrast-enhanced CT scan of the liver obtained during the hepatic arterial phase shows the tumor (arrow) as a heterogeneously enhancing mass in the hepatic dome. The tumor size was ~5.4 cm. (B) After five days of treatment, a CT scan of the liver obtained during the hepatic arterial phase at the same level as (A) shows that the tumor (arrow) enhancement areas decreased compared with the enhancement areas in (A). The tumor size was ~5.6 cm. CT, computed tomography.

**Figure 3:**
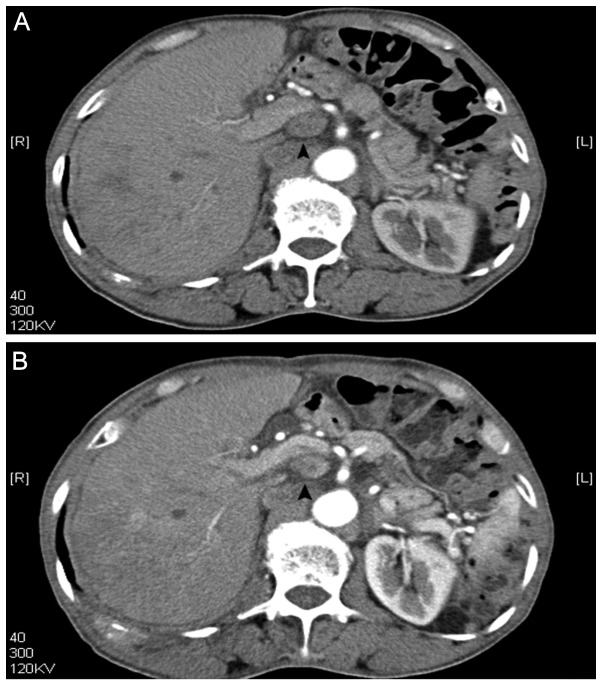
The size of the abdominal node decreased from 2.2 cm to 1.9 cm after ultrasound therapy. (A) Axial contrast-enhanced CT scan of the abdominal lymph node obtained during the hepatic arterial phase prior to treatment. (B) After five days of treatment, a CT scan of the liver obtained during the hepatic arterial phase at the same level as (A) shows that the size of the abdominal lymph node decreased compared with the scan in (A). CT, computed tomography.

A 71-year-old male presented to Nantong University Affiliated Nantong Tumor Hospital (Nantong, China) with abdominal pain and weight loss. Laboratory tests showed elevated carbohydrate antigen 19-9 (CA19-9) levels (2,007 U/ml; normal range, 0–39 U/ml) and negativity for hepatitis B virus-related antigens and antibodies. However, α-fetoprotein (1.5 ng/ml; normal range, 0–20 ng/ml), carcinoembryonic antigen (1.77 ng/ml; normal range, 0–5 ng/ml), alanine aminotransferase (25 U/ml; normal range, 4–40 U/ml), alkaline phosphatase (84 U/ml; normal range, 66–220 U/ml) and γ-glutamyl transferase (49 U/ml; normal range, 8–87 U/ml) were within the normal ranges. Imaging examinations including contrast-enhanced US (CEUS) (Fig. 1) and computed tomography (CT) (Fig. 2) identified a heterogeneously enhancing liver tumor in the hepatic arterial phase. A malignant tumor was suspected and an intrahepatic cholangiocarcinoma (ICC) was clinically diagnosed. The patient was not suitable for surgery, due to abdominal lymph nodes (Fig. 3) and spinal metastasis. Treatment with radiofrequency ablation was excluded, as the tumor was located in the posterior portion of the right lobe near the diaphragm. Doctors decided against transarterial chemoembolization, as ICC is not suitable for an arterial embolization (16). Therefore, the patient was offered other palliative therapy, such as low-frequency US. Prior to and following US treatment, CEUS and CT imaging, as well as the examination of CA19-9 levels, were used to evaluate the therapeutic effect. This study was approved by the human ethics committee of Nantong University Affiliated Nantong Tumor Hospital. Written informed consent was obtained from the patient’s family. Clinical data, laboratory tests, imaging data, US treatment and results were obtained from the patient’s medical records.

### Therapeutic set-up

**Figure 4:**
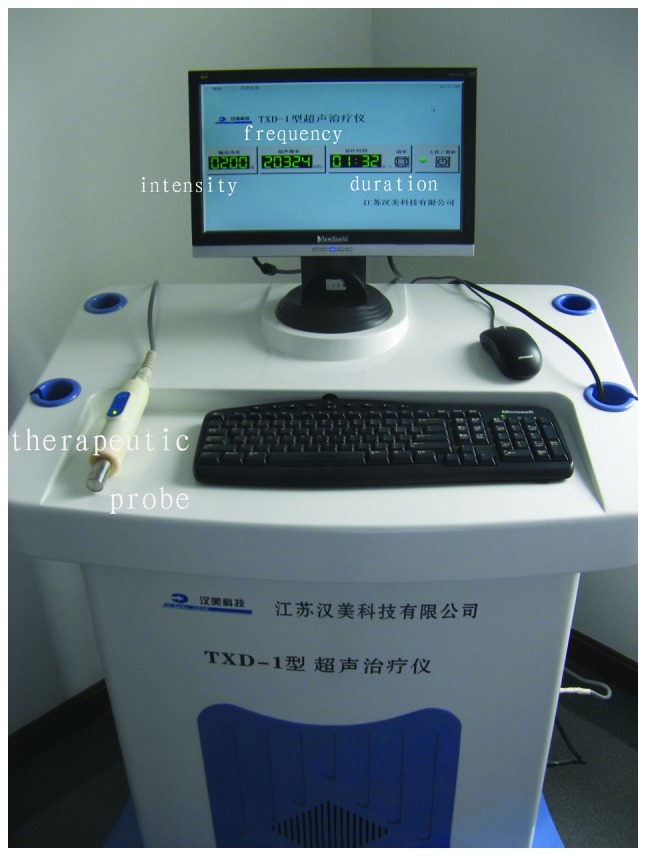
Low-frequency ultrasound apparatus.

**Figure 5:**
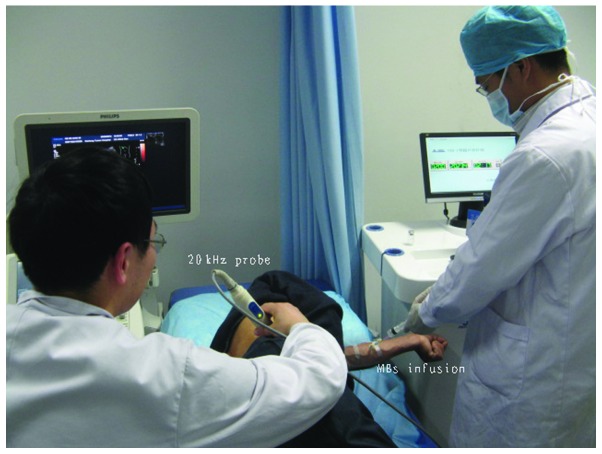
The tumor was exposed to 20 kHz ultrasound, and simultaneously infused with microbubbles through the ulnar vein.

The tumor was exposed to low-frequency US, which was accompanied simultaneously with MBs injected through the ulnar vein. The low-frequency US therapeutic unit (Fig. 4) was manufactured by the Jiangsu Hanmei Biology Technology Ltd., Co., (Taizhou, China). Prior to treatment, the ultrasonic instrument (iU22 US system; Philips Medical Systems, Bothell, WA, USA) was used to identify the hepatic tumor. The tumor was subsequently sonicated using a low-frequency US transducer. The diameter of the therapeutic US transducer was ~24 mm (Fig. 4). The probe was placed on the patient’s skin using US transmission gel (Aquasonic 100; Parker Laboratories, Inc., Fairfield, NJ, USA), which was interposed to ensure US propagation. The low-frequency US parameters were set at 20 kHz, 2 W/cm^2^, duty cycle 40% (on 2 sec, off 3 sec) for a duration of 5 min each day for a total of five days. MBs of the contrast agent were administered simultaneously with the ultrasonic irradiation by continuous infusion through the ulnar vein (Fig. 5). Each MB was composed of a phospholipid shell containing sulfur hexafluoride. A 5-ml dose of contrast agent was administered for each treatment at a concentration of 1.8×10^9^ microbubbles/ml.

### CEUS

CEUS is a useful method for assessing tumor neovascularity and monitoring anti-angiogenic therapies. In this study, the hepatic tumor was examined by CEUS after initiation and completion of treatment. CEUS images of the tumor were captured by an experienced examiner using the iU22 US system (Philips Medical Systems). The frequency of the probe was 3.5 MHz. Sulfur hexafluoride MBs (SonoVue; Bracco, Milan, Italy) were used. The agent (25 mg) was agitated for ~1 min with 0.9% saline solution (5 ml) and 2.4 ml suspension was injected manually as a bolus through a 5-ml syringe placed in the vein. Following injection of the bolus, the real-time enhancement pattern of the contrast agent was observed inside the tumor for 3–5 min and an imaging video was captured.

### CT scanning

Multiphasic CT was performed using a 64-section multidetector CT scanner (Somatom Sensation 64; Siemens Medical Solutions, Erlangen, Germany) with a gantry rotation speed of 500 msec, generator of 60 KW, pitch 1–1.5, 120 kVp and 320 mAs. The patient received a nonionic contrast medium [iomeprol, 400 mg of iodine per milliliter (Iomeron 400; Bracco Imaging, Milan, Italy)] at a dose of 1.3 ml (520 mg of iodine) per kilogram of body weight. The contrast medium was administered using a dual-chamber mechanical power injector (Stellant D CT; Medrad, Inc., Indianola, PA, USA) at a rate of 3 ml/sec through an 18-gauge intravenous catheter, which was inserted into an antecubital vein. This was followed by a 40-ml saline flush at the same injection rate.

### Results

The patient was treated in one session. Prior to treatment, the tumor size on CT was 5.4 cm. After treatment, the tumor size on the CT increased to 5.6 cm. After treatment with US and MBs, the intensity and enhanced-areas on the CEUS and the CT images were reduced (Figs. 1 and 2). Thus, insonation of tumors by US and MBs may be effective in reducing blood supply to tumors. After US treatment, abdominal lymph nodes decreased in size from 2.2 cm to 1.9 cm (Fig. 3), as shown by CT. The CA19-9 level decreased from 2,007 U/ml prior to therapy to 734 U/ml after therapy.

## Discussion

CEUS is useful for the evaluation of anti-angiogenic treatments. CT is also generally used for assessment of the response to anticancer therapy, as tumor size, necrotic lesions, vascularity and perfusion are evaluated simultaneously (17).

Although the tumor size, as shown on CT scans, increases marginally after treatment, the intensity-enhanced area of the tumor is decreased when compared with that prior to treatment. Another possible reason for the increased intensity-enhanced area observed in the tumor following therapy is that edema may have occurred (18,19).

After treatment, the tumor exhibits a poor parenchymal vascular network. Atrophy of the arterioles in the parenchyma may occur, and decreased blood perfusion may decrease the onset of tumor enhancement (14). These results lead to a reduction of the enhanced areas in the CEUS and CT scans in the arterial phase. Low-frequency US with a lower energy, such as a frequency of ~20 kHz, an intensity of 2 W/cm^2^, and a duration of ~5 min, may improve the efficiency of anti-angiogenesis therapy.

In the present study, the MBs were injected through the vein simultaneously with the US treatment. The MBs can function as cavitation nuclei, which reduce the threshold of US intensity required to induce bio-effects (20). Constrained within the blood vessels, the MBs excited by the US not only affect the vascular endothelium (21), but may also rupture the vessel (20,22). In a previous study, it was found that the confinement caused by the vessels and the surrounding tissue did not prevent the bubbles from undergoing large volumetric oscillations, which included inertial collapse. The blood vessels were deformed on the same microsecond time-scale as the bubble oscillations (21). Blood vessel deformations on μsec timescales caused by ultrasonic cavitation (23,24), including vessel distention, invagination and liquid jets, may contribute to vessel rupture. These deformations may also lead to a transient increase in temperature that alters the fluidity of the cell membrane (25). Local deposition of such high energy may result in cell damage that enhances the permeability of the endothelial cell layers (26). Another possibility is asymmetric bubble collapse and the formation of liquid jets (21). All three mechanisms, vessel distention, invagination and liquid jets, may contribute to vessel rupture (24). In our previous *in vivo* study, it was also found that 20 kHz US combined with MBs lead to vascular endothelial cell wall rupture, widened endothelial cell gaps and interstitial erythrocyte leakage in rabbit hepatic tumors (27). Following treatment, vessel rupture damaged the vascular network, decreased angiogenic activity, and decreased blood perfusion. These phenomena may explain why the onset of tumor enhancement (on CEUS and CT scans) was reduced.

In the present study, it was found that the antitumor treatment lead to a decrease in abdominal lymph node size on CT scans (Fig. 3). High-frequency US (10–12 MHz) has a superior resolution compared with that of low-frequency US on superficial organs, such as breast and thyroid. However, the penetration of this technique is inferior to that of low-frequency US. A total of 20 kHz US penetrates deep into tissues, while maintaining selectivity by being focused. The penetration of this method is better than that of higher frequency US. This presents a unique advantage of using 20 kHz US as a non-invasive treatment for deep tumors. The reason for the observed decrease in lymph node size may be that low-frequency US penetrates deeply into the tissues and focuses on the area of the abdominal lymph node. Furthermore, therapy appears to stimulate an immune response (28), which could thus inhibit lymph node growth.

In this study, the post-treatment level of CA19-9 was 734 U/ml, while the pre-treatment level was 2,007 U/ml. CA19-9 is a diagnostic and prognostic factor for ICC (29,30). The reduced levels of the serum marker indicated that the therapy used to treat the liver tumor was effective.

Approximately 70% of patients who present with hepatic malignant tumors are not eligible for curative treatment as they exhibit middle or advanced stage disease, and the prognosis is poor (31). In patients with tumors that exhibit a rich blood supply, anti-angiogenic treatment is required and will become widespread. Low-frequency US appears to present a potential minimally invasive and convenient method of tumor treatment. This modality may gain attention in the future as a minimally invasive method for the treatment of hepatic malignant tumors with a copious blood supply and with surgical contraindications. As low-frequency US remains a novel technology, to the best of our knowledge, this report is the first to investigate the effects of low-frequency US combined with MBs for the treatment of a patient with a hepatic malignant tumor. Future studies are required before low-frequency US can become a well-established method for tumor therapy.
